# Behavioral and Neurophysiological Effects of Transcranial Direct Current Stimulation (tDCS) in Fronto-Temporal Dementia

**DOI:** 10.3389/fnbeh.2018.00235

**Published:** 2018-10-29

**Authors:** Roberta Ferrucci, Simona Mrakic-Sposta, Simona Gardini, Fabiana Ruggiero, Maurizio Vergari, Francesca Mameli, Andrea Arighi, Marco Spallazzi, Federica Barocco, Giovanni Michelini, Anna Margherita Pietroboni, Laura Ghezzi, Giorgio Giulio Fumagalli, Giordano D'Urso, Paolo Caffarra, Elio Scarpini, Alberto Priori, Sara Marceglia

**Affiliations:** ^1^Fondazione Ca' Granda, IRCCS Ospedale Maggiore Policlinico, Milan, Italy; ^2^“Aldo Ravelli” Research Center for Neurotechnology and Experimental Brain Therapeutics, University of Milan Medical School, Milan, Italy; ^3^III Neurological Clinic, San Paolo Hospital, Milan, Italy; ^4^National Council of Research, Institute of Bioimaging and Molecular Physiology, Segrate, Italy; ^5^Department of Neuroscience, University of Parma, Parma, Italy; ^6^Department of Pathophysiology and Transplantation, “Dino Ferrari” center, University of Milan, Milan, Italy; ^7^Dementia Unit, Azienda Ospedaliero-Universitaria of Parma, Parma, Italy; ^8^Department of Neurosciences, Psychology, Drug Research and Child Health (NEUROFARBA), University of Florence, Florence, Italy; ^9^University of Naples “Federico II”-AOU, Naples, Italy; ^10^Center for Cognitive Disorders and Dementia, AUSL of Parma, Parma, Italy; ^11^Department of Engineering and Architecture, University of Trieste, Trieste, Italy

**Keywords:** transcranial direct current stimulation (tDCS), neuromodulation, fronto-temporal dementia, EEG, reaction time, neuropsychiatric inventory

## Abstract

Fronto-temporal dementia (FTD) is the clinical-diagnostic term that is now preferred to describe patients with a range of progressive dementia syndromes associated with focal atrophy of the frontal and anterior temporal cerebral regions. Currently available FTD medications have been used to control behavioral symptoms, even though they are ineffective in some patients, expensive and may induce adverse effects. Alternative therapeutic approaches are worth pursuing, such as non-invasive brain stimulation with transcranial direct current (tDCS). tDCS has been demonstrated to influence neuronal excitability and reported to enhance cognitive performance in dementia. The aim of this study was to investigate whether applying Anodal tDCS (2 mA intensity, 20 min) over the fronto-temporal cortex bilaterally in five consecutive daily sessions would improve cognitive performance and behavior symptoms in FTD patients, also considering the neuromodulatory effect of stimulation on cortical electrical activity measured through EEG. We recruited 13 patients with FTD and we tested the effect of Anodal and Sham (i.e., placebo) tDCS in two separate experimental sessions. In each session, at baseline (T0), after 5 consecutive days (T1), after 1 week (T2), and after 4 weeks (T3) from the end of the treatment, cognitive and behavioral functions were tested. EEG (21 electrodes, 10–20 international system) was recorded for 5 min with eyes closed at the same time points in nine patients. The present findings showed that Anodal tDCS applied bilaterally over the fronto-temporal cortex significantly improves (1) neuropsychiatric symptoms (as measured by the neuropsychiatric inventory, NPI) in FTD patients immediately after tDCS treatment, and (2) simple visual reaction times (sVRTs) up to 1 month after tDCS treatment. These cognitive improvements significantly correlate with the time course of the slow EEG oscillations (delta and theta bands) measured at the same time points. Even though further studies on larger samples are needed, these findings support the effectiveness of Anodal tDCS over the fronto-temporal regions in FTD on attentional processes that might be correlated to a normalized EEG low-frequency pattern.

## Introduction

Fronto-temporal dementia (FTD) is the clinical diagnostic term that is now preferred to describe patients with a range of progressive dementia syndromes associated with focal atrophy of the frontal and anterior temporal cerebral region (Piguet and Hodges, 2013). Epidemiological studies suggest that FTD is the second most common cause of young-onset dementia after Alzheimer's disease (AD) and accounts for 5–15% of all types of dementia (Seltman and Matthews, 2012).

Currently, available FTD medications have been used to control behavioral symptoms, even though they are ineffective in some patients, expensive and may induce adverse effects (Allain et al., 2003). Given this paucity of pharmacological interventions, strategies for non-pharmacological enhancement are receiving increasing attention, including the use of non-invasive stimulation, such as transcranial Direct Current Stimulation (tDCS), a neuromodulatory technique that delivers low-intensity direct current to cortical areas that facilitates or inhibits cortical spontaneous neuronal activity (Woods et al., 2016). Interesting findings have emerged in healthy volunteers and in clinical populations (Floel, 2014; Summers et al., 2016). Collectively, these studies have shown that tDCS is a safe tool able to enhance memory, language, attention, and learning processes (Shin et al., 2015). In clinical studies, previous findings in AD patients demonstrated that Anodal tDCS, both after a single session and after five consecutive daily sessions of tDCS over the temporal and parietal cortices, produces significant improvements in verbal and visual recognition memory (Ferrucci et al., 2008; Boggio et al., 2009, 2012) Notably, the tDCS effect persisted for at least 4 weeks after intervention.

Only few studies have tested the effects of tDCS treatment in FTD and the results are controversial. tDCS, usually applied bilaterally over the left inferior parieto-temporal region, provided encouraging results in treating anomia and other cognitive disabilities in demented individuals (Roncero et al., 2017) and in improving behavioral disturbances predominantly characterized by apathy (Agarwal et al., 2016), but failed to produce any improvement in behavioral and language function immediately after a single session of stimulation (Huey et al., 2007). Only one case-study reported the successful application of tDCS over 5 consecutive days that substantially improved behavioral disturbances and socio-occupational functioning in a woman with FTD (Agarwal et al., 2016). These results suggest that repeated tDCS sessions may be useful to enhance long-lasting tDCS effects, but need to be tested in larger samples.

As well as in other applications of non-invasive neuromodulation, the heterogeneity of stimulation protocols and the type of outcomes measured are among the major challenges to obtain consistent and comparable results (Elder and Taylor, 2014; Lefaucheur et al., 2017).

Recently, the use of quantitative electroencephalography (qEEG) to study the neurophysiological effects of tDCS showed that tDCS-induced modulations of EEG rhythms and coherences are consistent with the tDCS-induced effects on memory in patients with Alzheimer's Disease (Marceglia et al., 2016). The patients analyzed in Marceglia et al. (2016) were those described in a previous paper, in which the clinical effects of Anodal and cathodal tDCS applied bilaterally over temporo-parietal areas (P3-T5 and P4-T6 according to the international 10–20 EEG standard), with reference on the right shoulder, were studied (Ferrucci et al., 2008). Studying qEEG modifications in parallel with clinical and neuropsychological variables could hence strengthen the findings on the overall effects of tDCS. tDCS, in fact, could “normalize” the EEG pattern typical of the pathology under study (Koberda et al., 2013; Marceglia et al., 2016), thus providing both the neurophysiological basis of its positive effects on patients and a quantitative and repeatable outcome representative of the patient's state. Patients with cognitive decline are characterized by an increased power in the theta band (4–7 Hz) in fronto-temporal regions, and an overall decrease of beta power (13–35 Hz) with a focus in temporo-parietal areas (Koberda et al., 2013). In Alzheimer's disease, the abnormal beta pattern was reverted by Anodal tDCS, and tDCS-induced changes correlated well with the positive effects of the stimulation on working memory (Marceglia et al., 2016).

The purposes of this study were to investigate (1) whether applying Anodal tDCS over the frontal cortex in five consecutive daily sessions would improve cognitive performance and behavioral symptoms in FTD patients, and (2) whether these effects correlate with the neurophysiological pattern measured by EEG.

## Materials and methods

### Participants

We enrolled 13 patients diagnosed with FTD according to published criteria (Brun et al., 1994). Eight had the predominantly behavioral variant (3 female; mean age ± SD: 76.6 ± 0.57 years; 5 male; 69.4 ± 4.1 years) and five had the language variant (2 female; mean age ± SD: 73 ± 1.4 years; 3 male; 66.0 ± 3.6 years). Of these, one was excluded because did not complete the full study protocol. We therefore analyzed 12 subjects.

All patients were screened and recruited in the Center for Neurodegenerative Diseases at the Fondazione IRCCS Ca' Granda Ospedale Maggiore Policlinico, Milan, and at the Dementia Unit, Azienda Ospedaliero-Universitaria of Parma, Italy, by a team of experienced neurologists and neuropsychologists through appropriate diagnostic tests.

Participants were included in the study if their Mini Mental State Examination (MMSE) score was above 20 (mean ± SD: 24.4 ± 3.3) and if they had no other neuropsychiatric diseases. The demographic characteristics of the groups are summarized in Table 1. The patients were taking CNS-active medications and they maintained their medication regimen unchanged throughout the study (Table 1). Tau-protein measurements were collected (Table 1) and were in line with the available data for dementia patients (van Harten et al., 2011). CSF samples were obtained using a standardized protocol; lumbar punctures were performed in the mornings at L3/L4 or L4/L5 interspaced. About 1 ml of CSF was immediately frozen and stored at −80°C until biochemical assays for Tau-protein levels were performed. CSF levels of Tau-protein phosphorylated at threonine-181 were measured by ELISA, using a commercially available kit (Innotest PHOSPHO-TAU Antigen, Innogenetics, Belgium). The monoclonal antibodies which are coated on the ELISA plate recognize both the entire moiety and its fragments (Vanmechelen et al., 2000). Tau-protein values are expressed as pg/mL.

**Table 1:** Demographic and clinical features of the enrolled patients.

	**Education** **(Y)**	**MMSE**	**FTD** **variant**	**TAU** **protein pg/ml**	**Phosphorylates** **TAU protein pg/ml**	**Medication**
1	13	24	BV	138	38	Anti-hypertensive; Antidepressive
2	13	25	PPA	205	70	Anti-hypertensive; Antipsicotic; Cholinesterase Inhibitor; Insuline; Antiplatelet; Statins
3	13	21	BV	1,119	89	Anti-hypertensive
4	5	28	BV	32	17	Antipsicotic; Anti-hypertensive; Anxiolitic
5	13	23	BV	586	71	Antipsicotic Antidiabetic
6	18	22	PPA	1,005	95	Anti-depressive
7	18	27	PPA	580	61	Anti-hypertensive; Antiplatelet
8	13	30	BV	363	84	Anti-hypertensive; Anti-depressive Statins
9	8	20	BV	371	164	Statins Antidiabetic Antiplatelet
10	13	25	BV	313	35	Anti-hypertensive; Anti-depressive
11	8	28	PPA	211	81	Antiplatelet; Antipsicotic
12	5	30	BV	237	128	Anti-hypertensive; Anti-depressive; Statins
13	13	21	PPA	864	86	Antipsicotic

*MMSE, mini mental state examination; FTD, fronto-temporal dementia; BV, behavioral variant of FTD; PPA, primary progressive aphasia; SD, standard deviation; anti-hypertensive: nifedipine, candesartancilexetil, amlodipine, indapamide hemihydrates, hydrochlorothiazide/irbesartan, nebivolol; anti-depressive: citalopram; antipsicotic: quetiapine, promazine, haloperidol; cholinesterase inhibitor: rivastigmine; antiplatelet: cardioaspirine; statins: atorvastatin, antidiabetic: metformin; anxiolytic: hydroxyzine hydrochloride*.

The study was performed according to the Declaration of Helsinki and approved by the local institutional review board. Patients and their caregivers provided their informed and written consent before participation.

### Experimental protocol

We tested the effect of Anodal and Sham (i.e., placebo) tDCS applied daily to fronto-temporal lobes for 5 consecutive days in two separate experimental sessions. All subjects received both types of stimulation in a randomized and counterbalanced order (1:1 ratio). To avoid carry-over effects, an average of 60 ± 5 days elapsed between sessions. The patients and the examiner who performed the neuropsychological assessment were blind to the type of tDCS delivered in each session.

Cognitive functions and behavior were tested four times: at baseline (T0), after 5 consecutive days (T1), at 1 week (T2), and at 4 weeks (T3) after the end of the treatment. In addition, in 9 out of 13 patients, EEG was recorded four times, at the same time points (T0, T1, T2, T3).

### tDCS protocol

According to the available literature, tDCS was delivered bilaterally through a battery-driven constant current stimulator (HDCStim, Newronika srl, Milan, Italy) using three surface saline-soaked sponge electrodes, two placed on the scalp and one placed over the right deltoid muscle (each scalp electrode measured 35 cm^2^; the deltoid electrode measured 64 cm^2^). The rationale of bilateral stimulation is based on the fact that no asymmetry is expected in the areas that are treated, and, therefore, a unilateral stimulation would introduce an unwanted asymmetry, whereas bilateral stimulation would provide a balanced effect on both sides. The same stimulation protocol was proposed by Ferrucci et al. (2008) to treat Alzheimer's patients, but with a different electrode location.

**Figure 1:**
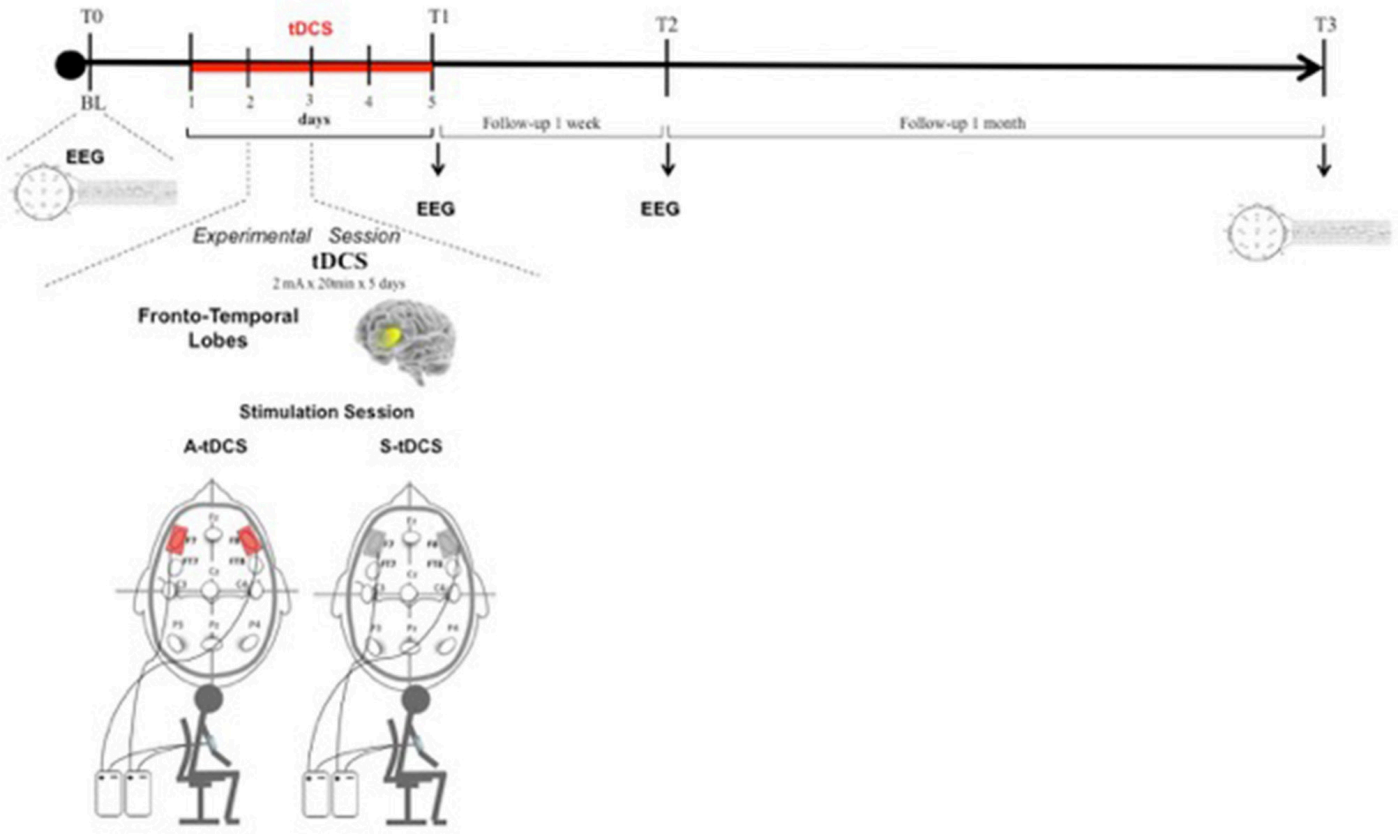
Transcranial Direct Current Stimulation (tDCS) experimental protocol. tDCS was applied bilaterally over the scalp on the Fronto-Temporal lobes for 5 consecutive days. Patients were assessed at baseline (T0), after 5 consecutive days of treatment (T1), after 1 week (T2), and after 4 weeks (T3) from the end of tDCS treatment.

Anodal stimulation consisted of 20 min of 2 mA direct current per session (with 10 s for ramping up and down) with the anode placed over the fronto-temporal lobes bilaterally (F7 and F8, according to the 10–20 EEG International System) and the reference electrode above the right deltoid muscle. The same procedure was used for Sham stimulation, but current was applied only for the first 10 s (Figure 1). To verify whether the patients could distinguish between active and Sham stimulation, we asked them to refer any sensation felt during tDCS sessions. They confirmed that in both cases they felt only the initial itching sensation disappearing after 10–20 s, without differences perceived between active and Sham stimulations.

### Cognitive and behavioral assessment

Cognitive functions were evaluated through five different tasks: the Phonemic Verbal Fluency Task (PFT) (Novelli et al., 1986), the Visual Recognition Task (VRT) (Boggio et al., 2012), the Picture Naming Task (PNT) (Viggiano et al., 2004), the Go no-Go Task (GGT) (Barbarotto et al., 1998) and, to investigate whether the effects of tDCS of cognitive performance can reflect changes in arousal, the Simple Visual Reaction Times Task (sVRT) (Barbarotto et al., 1998). Behavioral changes were evaluated with the Neuropsychiatric Inventory (NPI) (Cummings et al., 1994) and the Frontal Behavioral Inventory (FBI) (Kertesz et al., 1997). Furthermore, to evaluate the caregiver's burden, the Zarit Burden Inventory (ZBI) (Zarit and Zarit, 1990) was administered.

All the tests including possible biases due to learning were developed using variants, in order to avoid habituation and improvements due to the test replication, as described below.

PFT: this task was performed to measure the number of words beginning with a target letter that could be generated in 60 s, excluding proper nouns, numbers, and different forms of the same word. One letter for each condition was used, for a total of four letters (P, S, L, F) counterbalanced across stimulation conditions and order of presentation. The fluency score was the total number of words for each condition. Increased values indicate improvement.

VRT: in this task we specifically evaluated visual memory using a computer-controlled procedure.

The task comprised both encoding and recognition phases. It started with the encoding phase (two items), in which drawings of animals, persons, and objects were displayed on a computer screen for 10 s, followed 1 s later by the recognition phase, when patients were shown a single picture (test trial) and asked to say whether the picture had been presented before. Patients underwent this procedure eight times during the test. These eight encoding/recognition sequences included two study trials of two, four, six, and eight stimuli. Patients therefore studied a total 40 drawings during the test. Each study trial included test trials (recognition phase). Three test trials were presented after each two-item study trial; six test trials after each four-item study trial; eight test trials after each six-item study trial; and 10 test trials after each eight-item study trial. To avoid learning, we used alternative versions of this task and randomized them between assessment sessions. The memory score was the total number of items recognized for each condition (Boggio et al., 2012). Increased scores indicate improvement.

PNT: Subjects were asked to name pictures presented on a personal computer screen from one out of four lists (A–D). The lists were homogeneous for difficulties and were controlled for frequency of use, familiarity, visual complexity, grammatical class (nouns), and length in syllables; each list contained two items from a variety of semantic categories (living and non-living). Italian standardized norms for the name agreement and synonyms of the target word were accepted. The accuracy was the number of pictures correctly named in a 20-item list; we scored “1” for correct responses and “0” for errors. Increased values indicate improvement.

GGT: we administered this task to investigate response inhibition using a computer-controlled procedure (E-Prime-Psychology Software Tools, Inc.). Participants were required to look at a series of geometric figures, which could be either “square” or “circle,” randomly displayed on the screen and respond to a 35 target figure by pressing a button. The dependent variables measured to investigate response inhibition were RTs and accuracy (number of correct responses; Barbarotto et al., 1998). Decreased RTs and increased accuracy values indicate improvement.

sVRT: Thirty-five fully white squares appear one at a time on a PC screen at randomized intervals. The subject is asked to push down the space bar as quickly as possible after the stimulus appears. The median value of all the recorded time values is considered. The number of omissions is also registered (Barbarotto et al., 1998). Decreased values indicate improvement.

NPI: The NPI is a caregiver-based structured interview designed to briefly assess problematic behaviors and psychopathology in dementia. It evaluates 12 neuropsychiatric disturbances common in dementia: delusions, hallucinations, agitation, dysphoria, anxiety, apathy, irritability, euphoria, disinhibition, aberrant motor behavior, night-time behavior disturbances, and appetite and eating abnormalities. The severity and frequency of each neuropsychiatric symptom are rated on the basis of written questions administered to the patient's caregiver (Cummings et al., 1994). Decreased values indicate improvement.

ZBI: The caregiver's burden was evaluated using the 22-item ZBI (Zarit and Zarit, 1990). It consists of a semistructured questionnaire administered during the assessment interview and can be used to simultaneously evaluate both the material and emotional burden experienced by the caregiver. The scale is made up of 22 items evaluating disease impact on a caregiver's quality of life, psychological suffering, financial difficulties, Shame, guilt, and difficulties in social and family relationships. Scores range from 0 to 88. Decreased values indicate improvement.

FBI: The Frontal Behavioral Inventory (FBI) (Kertesz et al., 1997) is a 24-item caregiver questionnaire specifically developed to assess the behavioral disturbances of FTD. It has been shown to discriminate between different FTD phenotypes and between FTD and other forms of dementia. Decreased values indicate improvement.

### EEG recordings and analysis

EEG was recorded in a quiet room, with the subject awake, seated on a comfortable high-backed chair, under healthcare personnel continuous control, immediately after the administration of cognitive and behavioral tests. 21 electrodes (Ag/AgCl) were positioned according to the 10–20 International System using the EBNeuro Mizar-Light system (EBNeuro, Florence, IT). The average reference was used. The sampling frequency was 1,024 Hz with a bandpass of 0.5–500 Hz and a sensibility of 7 uV/mm. Signals were stored for further analysis. EEG was recorded for 5 min with eyes closed at the same time points used for neuropsychological and behavioral assessments: at baseline (T0), after 5 days of tDCS treatment (T1), after 1 week (T2), and after 4 weeks (T3) from the end of tDCS treatment.

The software toolbox EEGLAB, running under the cross-platform MATLAB environment (The Math-Works 7.0, Inc) was used for data processing. Preprocessing procedures included artifact rejection and filtering. EEG was analyzed in the frequency domain through parametric power spectrum estimation (Delorme and Makeig, 2004). Spectral power in the classical bands of EEG oscillatory activity, namely delta (1–3 Hz), θ (4–7 Hz), α (8–12 Hz), and β (13–35 Hz), was calculated for each subject below each electrode at each time point (T0, T1, T2, and T3).

We followed the same analysis methodology previously described in Marceglia et al. (2016) to assess EEG oscillatory activity in Alzheimer's disease. More specifically, as noted by Klimesch (1999), the exact definition of EEG frequency band can vary between subjects, and hence band powers should not be considered as fully independent variables. We therefore applied the same methodology as in Marceglia et al. (2016), and summed the contributions of delta and theta bands to cover the whole 2–7 Hz “low-frequency” range (i.e., the power for delta and theta was calculated separately and then summed), and summed the contributions of the alpha and beta bands to cover the whole 8–25 Hz “high-frequency” band, and focused our analysis on these two broad bands.

In addition, we divided the scalp into four regions of interest, namely frontal area (Fp1, Fp2, F3, F4, F7, F8), temporo-parietal area (T3, T4, T5, T6, P3, P4), central area (C3, C4), and occipital area (O1, O2). To obtain the low- and high- frequency power in each region of interest, we averaged EEG oscillations measured below each electrode belonging to the region. The right and the left areas were averaged, according to the assumption that no asymmetry is expected.

### Statistical analysis

To assess the neuropsychological and behavioral effects induced by tDCS, each test for cognitive functions (PFT, VRT, PNT, GGT), arousal (sVRT), behavioral changes (NPI and FBI) and caregiver's burden (ZBI) was analyzed independently. In addition, considering their non-continuous nature, we applied non-parametric statistics for clinical scales whereas parametric statistic was applied to continuous variables (such as reaction times). To account for the low number of subjects available, we ran two separate non-parametric one-way Friedman's ANOVAs with factor “time” (4 levels, T0-T3), one for the Anodal and one for the Sham tDCS session, and we corrected the overall result for these two comparisons (Bonferroni correction, *p* < 0.025). Then, to verify the effect at the single time points (whether existing) we applied *post-hoc* Wilcoxon signed ranked test with Bonferroni correction to take into account the effect of multiple comparisons (*p* < 0.01). We adopted the same analysis approach for continuous variables, but we used standard Bonferroni corrected one-way ANOVAs (*p* < 0.025) and Tukey's honest *post-hoc* test (*p* < 0.05) that already takes into account the effects of multiple comparisons (Cramer et al., 2016).

Finally, to have a direct comparison of the Anodal tDCS and Sham tDCS effects, we applied a two-way ANOVA with factors stimulation (2 levels, Anodal and Sham) and time (3 levels, T1-T3) on the changes from baseline of the clinical scales at T1, T2, and T3. For this analysis, to obtain the changes from baseline of clinical scales, we normalized the scale scores for the total of the scale as it follows:

$$\begin{array}{l} D_{\text{Tx}-\text{T}0}=\left(S_{\text{Tx}}-S_{\text{T}0}\right)/S_{\text{Tot}} \end{array}$$

Where D_Tx−T0_ is the change of the scale at the time point Tx with respect to T0, S_Tx_ is the score at Tx, S_T0_ is the score at T0, and S_Tot_ is the total value of the scale. Conversely, for continuous variables, such as reaction times, we calculated the percentage change from baseline as it follows:

$$\begin{array}{l} Dperc_{\text{Tx}}=\left(RT_{\text{Tx}}-RT_{\text{T}0}\right)/RT_{\text{T}0}^{*}100 \end{array}$$

Where RTperc_Tx_ is the percentage change from baseline of the continuous variable at the selected time point Tx, RT_Tx_ is value at Tx, and RT_T0_ is the value at T0.

Since we only wanted to verify whether there was any difference between the effects of Anodal and Sham tDCS on the clinical scales, regardless of the time, we considered this ANOVA as a planned comparison and only the factor stimulation was taken into account, thus allowing us not to correct the *p*-value (Cramer et al., 2016).

The analysis of EEG, considering that nine patients is a small sample size to obtain statistically relevant conclusions, is considered as an exploratory study and, therefore, only descriptive statistics are reported. We however wanted to verify whether there was a relationship between clinical outcomes significantly modulated by tDCS application and qEEG features. We considered as multiple predictors the values of the clinical scales that resulted significantly modulated by tDCS, and the qEEG power in each region of interest and in each band of interest as dependent variables.

We therefore applied separate multiple linear regression analyses between the power of each EEG band in each region of interest and the clinical scores at all time points (T0-T3), including only those scales identified as significant by the previous statistical analysis.

## Results

### Neuropsychological and behavioral effects of tDCS

To evaluate the effects of Anodal and Sham tDCS on neuropsychological and behavioral variables, we first assessed whether their time course showed significant changes in the two sessions. We found that, whereas Anodal tDCS significantly improved NPI scores and sVRTs, Sham tDCS failed to induce changes in the outcomes of these tests after its application.

**Figure 2:**
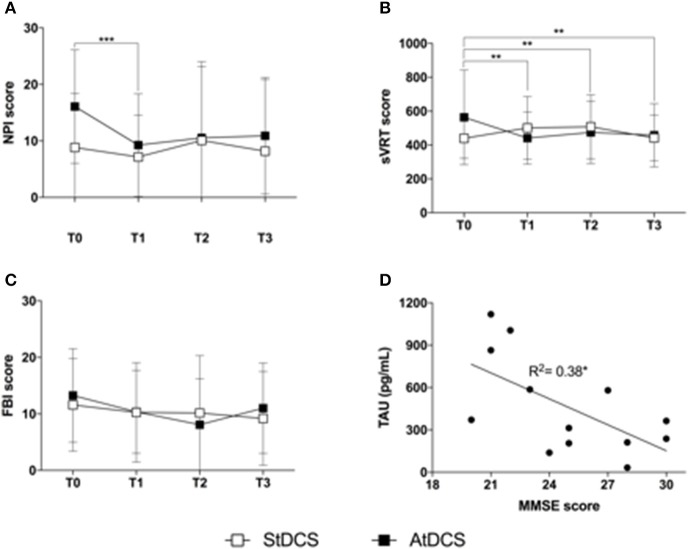
Findings on clinical variables **(A)**. Effect of Anodal (black squares) and Sham (white squares) tDCS on the Neuropsychiatric Inventory (NPI). Squares represent the average NPI score on the 12 subjects analyzed, at T0, T1, T2, and T3. Error bars are standard deviations. ^***^p < 0.01 at the *post-hoc* Wilcoxon signed ranked test with Bonferroni correction (significant); **(B)**. Effect of Anodal (black squares) and Sham (white squares) tDCS on the simple Visual Reaction Time (sVRT) test. Squares represent the average sVRT score on the 12 subjects analyzed, at T0, T1, T2, and T3. Error bars are standard deviations. ^**^p < 0.01 at the *post-hoc* Wilcoxon signed ranked test with Bonferroni correction (significant) **(C)**. Effect of Anodal (black squares) and Sham (white squares) tDCS on the Frontal Behavioral Inventory (FBI-A). Squares represent the average FBI-A score on the 12 subjects analyzed, at T0, T1, T2, and T3. Error bars are standard deviations **(D)**. Scatter plot of the correlation between TAU protein (pg/mL) and MMSE score. The line represents the estimated linear regression. ^*^p < 0.05.

More specifically, the non-parametric Friedman's ANOVA showed significant differences across time in NPI scores (Figure 2A) after Anodal tDCS (*p* = 0.006) but not after Sham tDCS (*p* = 0.11). *Post-hoc* analysis highlighted a significant decrease of NPI scores at T1 as compared to T0 after Anodal tDCS (T0 vs. T1: 16.09 ± 2.76 vs. 9.27 ± 2.50, *p* = 0.0077), and a tendency to decrease at T2 and T3 as compared to T0 (vs. T2: 10.55 ± 3.48, *p* = 0.047; vs. T3 10.91 ± 2.84, *p* = 0.075). This differential effect of tDCS was confirmed by the comparative analysis between changes from baseline after Anodal and Sham tDCS, that showed a significant effect of the “stimulation type” (*p* = 0.034). Because, as shown in Figure 2A, the NPI score at T0 in the Sham condition is less, on average, than in the Anodal condition, we ran a Wilcoxon signed rank test between baseline (T0) values in the two tDCS conditions, and found that there is no statistical difference (T0 Sham vs. T0 Anodal: 8.83 ± 9.15 vs. 16.05 ± 9.59, *p* = 0.075).

Also sVRTs (Figure 2B) were improved by Anodal tDCS (ANOVA *p* = 0.025) but not by Sham tDCS (ANOVA *p* = 0.15). *Post-hoc* analysis showed a significant decrease of sVRTs at T1, T2, and T3 as compared to T0 after Anodal tDCS (T0 vs. T1 671.59 ± 132.1 vs. 488.46 ± 65.32, *p* = 0.002; T2: 501.62 ± 57.22, *p* = 0.003; T3: 465.63 ± 49.34, *p* = 0.005). The comparative analysis of percentage changes after Anodal and Sham tDCS confirmed this observation (ANOVA factor “stimulation type,” *p* = 0.046).

No significant changes were observed in the other neuropsychological measures for both stimulation types. Figure 2C reports the behavior of FBI-A scores in the two stimulation conditions, that showed a tendency toward improvement after Anodal tDCS (non-parametric ANOVA *p* = 0.057).

Finally, we found a relationship between TAU protein (pg/mL) and MMSE score (Spearman's correlation coefficient *R*^2^ = 0.32, *p* = 0.05, Figure 2D).

### Correlation between clinical and qEEG effects

Table 2 reports the detailed descriptive statistics of LF and HF band power in all the different regions of interest during the Anodal and Sham tDCS sessions. LF power shows a decreasing behavior more marked after Anodal than after Sham tDCS in the Frontal and Temporo-Parietal areas, thus supporting the hypothesis that Anodal tDCS improves the bioelectrical pattern of FTD patients. Conversely, it seems that the effect on HF band power is similar between Anodal and Sham tDCS, with a general decreasing behavior over time.

**Table 2:** qEEG power in the low- and high- frequency bands at all time points and in all regions of interest.

**Area**			**S-tDCS**					**A-tDCS**			
			**T0**	**T1**	**T2**	**T3**		**T0**	**T1**	**T2**	**T3**
**FRONTAL**	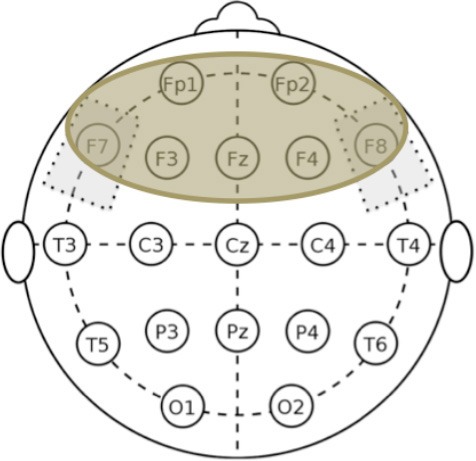	LF	14.86 ± 13.08	9.53 ± 10.90	10.36 ± 4.88	7.95 ± 5.95	LF	26.76 ± 32.74	20.54 ± 27.21	28.15 ± 30.87	14.35 ± 11.07
		HF	5.42 ± 1.68	3.40 ± 1.62	3.87 ± 0.85	4.85 ± 1.59	HF	5.98 ± 2.81	4.80 ± 1.68	4.85 ± 1.84	5.14 ± 2.09
**TEMPO-PARIETAL**	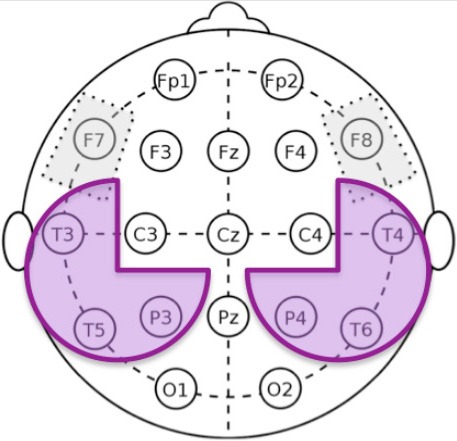	LF	30.41 ± 26.43	19.42 ± 22.31	18.13 ± 9.26	14.78 ± 9.55	LF	52.38 ± 71.09	41.99 ± 57.71	58.72 ± 66.36	27.13 ± 26.02
		HF	9.48 ± 2.29	6.98 ± 3.70	6.42 ± 2.30	7.44 ± 2.61	HF	19.45 ± 18.28	12.65 ± 7.42	14.23 ± 8.76	15.77 ± 7.73
**CENTRAL**	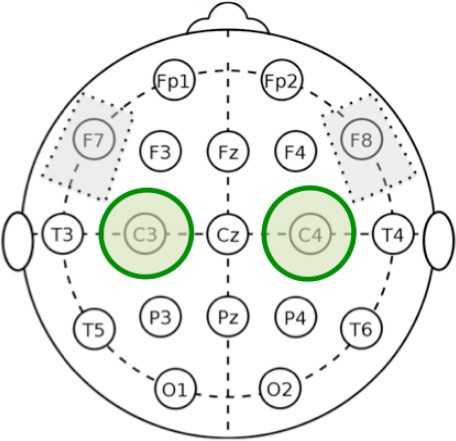	LF	27.12 ± 22.98	18.09 ± 17.41	15.74 ± 12.74	10.44 ± 10.45	LF	49.22 ± 69.20	44.15 ± 60.68	48.57 ± 60.68	19.39 ± 22.18
		HF	9.69 ± 6.13	7.79 ± 4.25	6.00 ± 1.28	5.34 ± 4.48	HF	12.18 ± 8.52	10.37 ± 9.31	10.04 ± 10.25	9.62 ± 7.09
**OCCIPITAL**	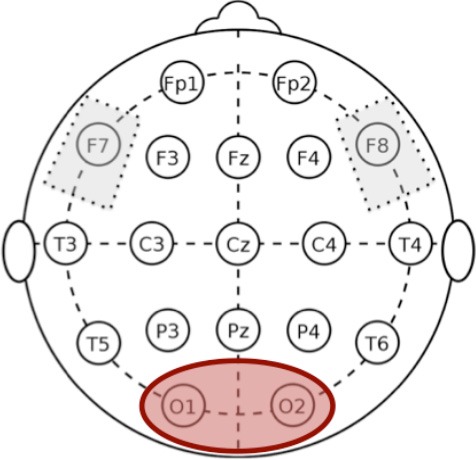	LF	48.46 ± 29.43	39.86 ± 30.02	46.13 ± 37.49	29.64 ± 34.11	LF	89.11 ± 107.82	70.74 ± 82.90	86.16 ± 105.10	40.18 ± 42.29
		HF	30.56 ± 18.28	29.06 ± 24.48	27.99 ± 39.35	25.35 ± 45.79	HF	59.75 ± 90.60	32.34 ± 28.84	35.23 ± 33.71	32.05 ± 34.33

**Figure 3:**
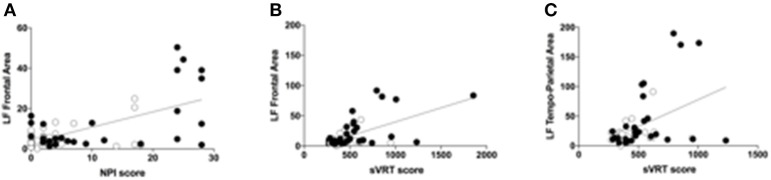
Correlations between qEEG and clinical variables **(A)**. Scatter plot of the correlation between the low-frequency (LB) band power calculated in the frontal area and the NPI scores. Black circles represent the values during the Anodal tDCS session, while the white circles represent the values during the Sham tDCS session. The scatter plot represents all the values at all time points (T0, T1, T2, and T3) **(B)**. Scatter plot of the correlation between the low-frequency (LB) band power calculated in the frontal area and the sVRT scores. Black circles represent the values during the Anodal tDCS session, while the white circles represent the values during the Sham tDCS session. The scatter plot represents all the values at all time points (T0, T1, T2, and T3) **(C)**. Scatter plot of the correlation between the low-frequency (LB) band power calculated in the temporo-parietal area and the sVRT scores. Black circles represent the values during the Anodal tDCS session, while the white circles represent the values during the Sham tDCS session. The scatter plot represents all the values at all time points (T0, T1, T2, and T3).

As shown in Figure 3, LF power in the Frontal area is significantly correlated to both NPI (*b* = 0.779, *p* = 0.009) and sVRTs (*b* = 0.43, *p* = 0.001), and LF power in the Temporo-Parietal area is correlated to sVRTs (*b* = 0.36, *p* = 0.003) thus suggesting that the tendential improvement in the EEG pattern is consistent with the observed clinical improvement in these patients. Conversely, LF power in the Central and Occipital areas, as well as HF power in all the regions of interest did not significantly correlate with clinical outcomes.

## Discussion

Our findings showed that Anodal tDCS over the fronto-temporal cortex improves both processing speed, as measured by simple reaction times, and the neuropsychiatric symptoms of dementia, as measured by the NPI scores, in FTD patients. The improvements observed were registered immediately after the end of the treatment and tended to be maintained after 1 week and 1 month. Also, the time course of the clinical measurements correlated with the time course of the neurophysiological qEEG pattern, showing a tendency toward normalization of LF activity which is known to be abnormally increased in dementia patients (Koberda et al., 2013).

The sVRT paradigm has been extensively used to measure processing speed and to evaluate attentional functions, and it is considered to be a suitable measure of dementia risk (Kochan et al., 2016). Indeed, people with AD present a slower reaction time, as well as prodromal individuals with Mild Cognitive Impairment (MCI) (Gorus et al., 2008). FTD patients tend to be slower than healthy controls in the RT paradigm, showing abnormal attentive processes related to frontal lesions. Manenti et al. (2015) found a reduction of vocal RTs during action naming after Anodal tDCS over the parietal cortex in a sample of patients with corticobasal syndrome, which is a neurodegenerative disorder that overlaps both clinically and neuropathologically with FTD (Manenti et al., 2015). Whereas, the choice of the tDCS target by Manenti et al. (2015) was based on the results on AD patients, which are usually stimulated over parietal or temporo-parietal areas, our tDCS target choice was based on the known characteristics of brain areas impairments in FTD patients. In fact, our sample encompasses a heterogeneous group of FTD conditions and can be broadly divided into behavioral variant fronto-temporal dementia (bv-FTD) and primary progressive aphasia (PPA). Bv-FTD is associated with predominant atrophy in the frontal and paralimbic areas, while PPA is commonly associated with temporal atrophy. We therefore chose to stimulate the fronto-temporal areas bilaterally. Furthermore, we used the Simple RTs task to measure general alertness and motor speed while Manenti et al. used a vocal reaction times to measure naming performance, and, despite different protocols, we obtained similar results. A reduction of RTs (perceptuo-motor vs. the verbal task) provide further evidence of the relationship between action and language. This fits in well with the perception-for-action-control theory (PACT) (Schwartz et al., 2002), stating that the perceptuo-motor links contribute to co-structuring of perceptual and motor representations and to perceptual organization of speech (Basirat et al., 2012).

Reaction Time is an important factor in relation to the integrity and efficiency of brain functions, such as those involved in attention, cognition, and perception. It has been defined as a behavioral “marker” of neurophysiological integrity (Haworth et al., 2016) and it might provide a “real-life” indicator of changes to everyday functions. RTs studies allow measuring other parameters, such as fatigue, stimuli and threshold responses, processing load, resource availability and utilization, patterns of functional decline and integrity, and response to interventions.

The loss of white matter integrity is associated with a disproportionate slowing of RTs (Kerchner et al., 2012). In particular, cognitive processing speed is related to the integrity of the frontal lobe (Kochunov et al., 2010).

The reduction of RTs in FTD patients observed in this study after Anodal tDCS might represent a cognitive marker of increased functional integrity (i.e., normal functioning) (Phillips et al., 2013) in these patients. In fact, because excitability alterations have been shown to have a specific effect on RT task performance (Nitsche et al., 2003; Antal et al., 2004; Wade and Hammond, 2015), our findings suggest that the cortical excitability changes induced by tDCS can improve cerebral integrity. The improvement of this cognitive index was accompanied by a reduction of the neuropsychiatric symptoms of dementia (NPI scores). In contrast with our results, Huey et al. (2007), studying 10 FTD patients receiving single sessions of unilateral Anodal and Sham tDCS in the frontal areas (above F3 electrode in the international 10–20 system), found no effects of Anodal tDCS on NPI scores. The differences could depend on methodological issues, including the duration, type and site of stimulation. In fact, we applied tDCS bilaterally over the fronto-temporal areas for 5 consecutive days, thus suggesting that a longer exposure to tDCS might be more effective than the application of a single session (Lefaucheur et al., 2017)

The FTD patients involved in the present study displayed prominent apathy that is the most common neuropsychiatric symptom associated with FTD. The behavioral and biological mechanisms of apathy, however, are not well-understood. Massimo et al. (2015) hypothesized that goal-directed behavior is supported by a network of multiple frontal brain regions. Overall, data from studies on psychiatric disorders suggest that tDCS over the dorsolateral prefrontal cortex (DLPFC) (Brunoni et al., 2010, 2011; Kuo et al., 2014; D'Urso et al., 2017, 2018) has the potential to induce clinically relevant behavioral changes in difficult-to-treat patient populations and could thus represent a valuable tool for intervention in a range of mental and neurological disorders.

Conversely, our findings of no tDCS-induced effects on language and verbal fluency confirms that of Huey et al. (2007). In fact, they did not find any effect of tDCS in improving verbal fluency. The authors proposed that this negative result may have been due to the fact that the stimulation session was not coupled with language therapy (Huey et al., 2007). Other studies that did not couple tDCS with language therapy have repeatedly yielded no improvement in both healthy and patient populations (Antal et al., 2007; Segrave et al., 2014). In contrast, Cotelli et al. (2014) found a beneficial effect of language training in combination with brain stimulation in PPA patients (Cotelli et al., 2014). Furthermore, the lack of language improvement could depend on sample characteristics (ceiling effect in linguistic tasks), given that most patients had predominantly behavioral symptoms.

Finally, the improvement in RT performance and NPI scores correlated with the qEEG pattern in the LF band that showed a tendency to decrease after Anodal tDCS. The abnormal increase of LF activity is suggested to be associated to Alzheimer's Disease (Duffy et al., 1984; Chiaramonti et al., 1997; Jelic et al., 2000; Kramer et al., 2007; Koberda et al., 2013; Fonseca et al., 2015), and, more specifically, to the slower information encording processes in these patients (Klimesch, 1999).

Despite exploratory, these results on the correlation between clinical and neurophysioogical variables suggests that studying qEEG features could help complementing clinical findings, especially in small groups of patients, by showing a tendency to improvements in the general brain state of the patients undergoing tDCS treatment.

This study was limited by the low sample size, which did not allow to run a full statistical comparison, especially for evaluating the effects of Active vs. Sham tDCS in time, and for comparing tDCS effects over the different variances of FTD. Further studies on a larger sample of FTD patients considering the different variants may be useful in understanding the maintenance effect of cognitive and behavioral improvement associated with fronto-temporal Anodal tDCS.

Altogether, these findings support the effectiveness of Anodal tDCS over the fronto-temporal regions in FTD on attentional processes, and suggest that tDCS-related improvements are related to a normalization of low frequency oscillations at the frontal and temporo-parietal levels.

## Author contributions

RF, SM-S, ES, AP, and SM: design/conception; RF, SM-S, SG, FB, MV, FM, AA, MS, FB, GM, AP, LG, GF, GD, PC, ES, AMP, and SM: literature and database search; SM, SM-S, and RF: data analysis; RF, SM-S, SG, FB, MV, FM, AA, MS, FB, GM, AP, LG, GF, GD, PC, ES, AMP, and SM: writing the initial draft of the manuscript; All authors critically revised and approved the final manuscript.

### Conflict of interest statement

RF, SM-S, MV, FM, AP, and SM are stakeholders in Newronika s.r.l., a spin-off company formed by the Fondazione IRCCS Ca' Granda Ospedale Maggiore Policlinico and Università degli Studi di Milano, Italy. The remaining authors declare that the research was conducted in the absence of any commercial or financial relationships that could be construed as a potential conflict of interest.
